# Usutu Virus in Bats, Germany, 2013

**DOI:** 10.3201/eid2010.140909

**Published:** 2014-10

**Authors:** Daniel Cadar, Norbert Becker, Renata de Mendonca Campos, Jessica Börstler, Hanna Jöst, Jonas Schmidt-Chanasit

**Affiliations:** Bernhard Nocht Institute for Tropical Medicine, Hamburg, Germany (D. Cadar, J. Börstler, H. Jöst, J. Schmidt-Chanasit);; German Mosquito Control Association, Speyer, Germany (N. Becker);; Federal University of Rio de Janeiro, Rio de Janeiro, Brazil (R. de Mendonca Campos);; German Centre for Infection Research, Hamburg (H. Jöst, J. Schmidt-Chanasit)

**Keywords:** Usutu virus, viruses, full-length genome, *Pipistrellus* bat, phylogeny, Germany

**To the Editor:** Usutu virus (USUV) is an arthropod-borne flavivirus that belongs to the Japanese encephalitis serocomplex (*1*). USUV circulates between ornithophilic mosquito vectors (mainly *Culex* spp. mosquitoes) and avian amplification hosts (*2*). Migratory birds play a key role in the introduction of USUV into new areas (*3*). USUV has recently been introduced from Africa into Europe, causing epizootics among wild birds and Usutu fever in humans (*4*–*6*). The detection and isolation of USUV from different bird and mammalophilic mosquitoes during the epizootic in Germany raise questions regarding the USUV host range (*2*,*3*). Bats have been considered natural reservoir hosts of a wide diversity of viruses, including several flaviviruses (*7*,*8*). Their ability to fly and their social behavior enable efficient maintenance, spread, and evolution of viruses. 

In September and October 2013, in southwest Germany, 2 dead bats were found within ≈15 km of each other (bat 1, Ludwigshafen am Rhein, 49°28′34′′N 8°26′46′′E; bat 2, Waldsee, 49°23′44′′N 8°26′27′′E), corresponding to the previously described USUV-endemic area (*2*,*3*). A full necropsy was conducted on each bat, and samples were collected for virus detection, histologic analysis, and bat species determination. 

Total DNA and RNA were extracted from tissue samples (brain, liver, lung, and heart) and subjected to reverse transcription PCR for rhabdovirus and flavivirus (*2*). Histologic analysis of the tissue samples was not successful because of autolysis. Use of a cytochrome b–specific PCR and direct sequencing of the PCR amplicons genetically identified each bat as a common pipistrelle (*Pipistrellus pipistrellus*) (*9*).

The bat samples were negative for rhabdoviruses but positive for flaviviruses (brain tissue only). Direct sequencing of the PCR amplicons revealed that the USUV sequences were related to the recently described bird-derived USUV strain BH65/11–02–03 from Germany (*2*). Attempts to isolate the bat USUV strains in cell culture were not successful, probably because of autolysis. However, the complete genome sequences of both bat USUV strains (BAT1USUTU-BNI, KJ859682; BAT2USUTU-BNI, KJ859683) were then determined directly from the brain samples by using primers (Technical Appendix) designed from multiple alignments of USUV genomes obtained from databases. 

The 2 genomes had an identical size of 11,065 nt with a 96-nt 5′ nontranslated region and a 664-nt 3′ nontranslated region. The single open reading frame encodes a polyprotein of 3,434 aa. Both bat USUV strains had 99.9% nt and 99.8% aa identity. We compared the 2 bat USUV strains with those detected in mosquitos, birds, and humans from Europe and Africa; the sequence identities of nucleotides varied from 78.3% to 99.3% and of amino acids from 90.8% to 99.3%. The sequence identity matrix with the USUV strain BH65/11–02–03 from Germany was 99.3% for nucleotides and 99.2% for amino acids. Comparison of the *Pipistrellus* bat USUV complete polyprotein sequence with mosquito and bird-derived strains showed 2 aa substitutions—one (A1236V) in the nonstructural protein (NS) 2a and the other (L1549F) in the NS3 gene—which were detected only in the bird-derived USUV strain BH65/11–02–03 from Germany. In addition, 2 additional unique amino acid substitutions (A1841V and K1870M) in the NS3 protein gene of the BAT1USUTU-BNI strain were also identified. Bayesian and maximum-likelihood phylogenetic analyses of the full-length sequences revealed the close relationship of the *Pipistrellus* bat–derived USUV strains with the 2011 bird-derived strain BH65/11–02–03 from Germany, forming a distinct group within the phylogenetic tree (group Europe 3) (Figure). A partial envelope and NS5-gene–based phylogenetic analysis that used more available sequences from databases yielded the same topology (data not shown). 

**Figure:**
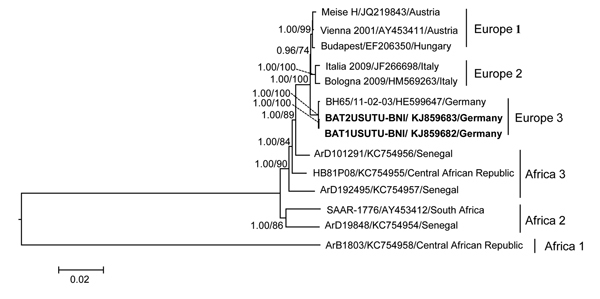
Maximum-likelihood phylogenetic tree of *Pipistrellus* bat Usutu viruses (USUV) based on full-length nucleotide sequences and showing the phylogenetic placement of the bat-derived USUV compared with human-, mosquito-, and bird-derived strains. The phylogenetic analyses were performed by using PhyML 3.0 (http://www.atgc-montpellier.fr/phyml/versions.php) with 1,000 pseudo-replicates and parallel Bayesian Markov chain Monte Carlo tree-sampling methods based on 2 runs consisting of 4 chains of 1,000,000 with a burn-in of 25% using MrBayes 3.1.2 (http://mrbayes.sourceforge.net/). The Akaike information criterion was chosen as the model selection framework and the general time-reversible model of sequence evolution with gamma-distributed rate variation among sites as the best model. Numbers at the nodes indicate maximum-likelihood bootstrap replicates (>70%) and parallel the posterior probability values (clade credibilities >90%). Boldface indicates USUV strains from *Pipistrellus* bats in Germany in 2013 (this study). Strain names, GenBank accession numbers, and countries of origin for sequences used to construct the tree are indicated on the branches. Scale bar indicates mean number of nucleotide substitutions per site.

*Pipistrellus* bats are highly prevalent in Germany. Their geographic range overlaps with that of the USUV epizootic. Thus, considerable interactions between birds, mosquitoes, and bats could have occurred. The amino acid replacements (A1236V and L1549F) detected in the NS genes of *Pipistrellus* bat–derived USUV strains and the bird-derived USUV strain from Germany suggest an adaptive evolution, which probably occurred during the introduction of the virus into Germany. 

Although the role of these mutations is not known, similar mutations in the related West Nile virus modulated the host antiviral response by inhibition of interferon signaling (*10*). Our results suggest that bats probably contribute to the epizootic rather than act as a silent reservoir for the virus. In contrast, infections of bats might be merely coincidental to what may well be broader infections of vertebrates in the epizootic area. However, for confirmation of this hypothesis, further investigations are required. Although the detected bat-derived sequences are somehow distinct from sequences of other USUV strains, a spillover infection from birds or another, yet unrecognized, host cannot be ruled out. The detection of the virus exclusively in brain tissue suggests that USUV might have a higher tropism for the nervous system in bats, as opposed to the pantropism observed in birds (*2*). The detection of USUV in bats raises questions for further research, including the potential role of bats as reservoirs in Africa and transmission by mosquito vectors.

Technical Appendix List of primers used for the full-length amplification of Usutu virus.
