# Supplementary material for: Usutu Virus in Bats, Germany, 2013
Source: Emerg Infect Dis. 2014 Oct;20(10):1771–3. doi: 10.3201/eid2010.140909 (PMC4193186; doi:10.3201/eid2010.140909)
Supplement: Technical Appendix — List of primers used for the full-length amplification of Usutu virus. [file 14-0909-Techapp-s1.pdf]

DOI: http://dx.doi.org/10.3201/eid2010.140909

Usutu Virus in Bats, Germany, 2013

Technical Appendix

Technical Appendix Table. Primers used for the full-length amplification of Usutu virus

Name	Primer sequence	Position	Length
USUV_GF	5′-AGWYGTTSGYCTGYGTGAGC-3′	1-740	740
USUV_731R*	5′-CGCTTCGAGTGTCTGGTTCT-3′		
USUV_8F	5′-CGTCTGCGTGAGCTCTACTACTTA-3′	8-400	393
USUV_400R	5′-TTTTTGTGCCCCGATTGTT-3′		
USUV_52F	5′-TGAGATTAACACAGTGCCGG-3′	52-832	781
USUV_832R	5′-ATCTCGAAGCCTTGGTTGAG-3′		
USUV_712F	5′-AGGTGCACAAGAACCAGACA-3′	712-1727	1016
USUV_1727R	5′-GATTGCTTTGTGGCATGGGG-3′		
USUV_1560F	5′-GTTGAACACCGAGGCATACTACAT-3′	1560-2461	902
USUV_2434R	5′-TGGCGAGAAAGAGGAGCAC-3′		
USUV_2356F	5′-CAGGGTCTAATGGGAGCTCT-3′	2356-3320	965
USUV_3320R	5′-CCTGGGCAATAGTCAAAGTC-3′		
USUV_3241F	5′-CGGCGTGAAGGTTACAAAGT-3′	3241-4160	920
USUV_4160R	5′-GAGAACTGCCCTGTTGATGT-3′		
USUV_3776F	5′-CCTTCAAAATTCAACCAGGC-3′	3776-4778	1001
USUV_4778R	5′-ATAGCTGCCCCTCTTGTGGT-3′		
USUV_4541F	5′-GGACACCATGGGCAATAATACCT-3′	4541-5872	1332
USUV_5872R	5′-CTCTGCTGGCCCCAAAGTTCG-3′		
USUV_5669F	5′-ACACCGGGAAAACAGTCTGG-3′	5669-6802	1134
USUV_6802R	5′-TGAGCAGAGCCAGCAATARR-3′		
USUV_6661F	5′-GTTTTCTTGCTCCTCGTTCA-3′	6661-7457	797
USUV_7457R	5′-CCAATCAGTAAAATCTGGCC-3′		
USUV_7368F	5′-GGTAGATGGTTTGGTGGCTA-3′	7368-8160	793
USUV_8160R	5′-TTGTTCTTCCACCTCAGCAC-3′		
USUV_8038F	5′-TATGGCTGGAACCTTGTCAC-3′	8038-9057	1020
USUV_9057R	5′-CCCCATCATGTTGTAAATGC-3′		
USUV_8987F	5′-AAATGGTGGACGAAGAAAGG-3′	8987-9865	879
USUV_9865R	5′-TCCTTCCGTCCTTCATGATC-3′		
USUV_9643F	5′-GAGAACGGAGAAGAAAGGGT-3′	9643-10823	1181
USUV_10823R	5′-AACAGTTCGCATCACCGTCT-3′		
USUV_10673F	5′-GGGACCCTGCCTATTGG-3′	10673-11027	355
USUV_11027R	5′-GCGCTCTGTGCCTTGTGGTTGAT-3′		
USUV_ADF	5′-GAAAGCCCCTCAGAACCGTTTC-3′	10646-11066	420
USUV_11014R*	5′-AGATCCTGTGKTCTWSYYCMCCAYCAG-3′		

*Nucleotide positions are according to the Vienna strain genome (GenBank accession no. AY453411) (*1*).

Reference

1. Bakonyi T, Gould EA, Kolodziejek J, Weissenböck H, Nowotny N. Complete genome analysis and molecular characterization of Usutu virus that emerged in Austria in 2001: comparison with the South African strain SAAR-1776 and other flaviviruses. Virology. 2004;328:301–10. **PMID: 15464850**
